# Delays in presentation, diagnosis, and treatment in Sudanese women with breast cancer: a cross-sectional study

**DOI:** 10.1093/oncolo/oyae066

**Published:** 2024-04-20

**Authors:** Esraa S A Alfadul, Badria Tebaig, Salma S Alrawa, Ammar Tarig Elgadi, Ensaf E M A Margani, Maab E B Adam, Mawaheb Sh Adem Mohamoud, Safa A M Elhassan, Moawia Mohammed Ali Elhassan

**Affiliations:** Faculty of Medicine, University of Khartoum, Khartoum, Sudan; Station of Medical Essentials (SOME) Institution, Khartoum, Sudan; Faculty of Medicine, University of Khartoum, Khartoum, Sudan; Faculty of Medicine, University of Khartoum, Khartoum, Sudan; Faculty of Medicine, University of Khartoum, Khartoum, Sudan; Station of Medical Essentials (SOME) Institution, Khartoum, Sudan; Faculty of Medicine, University of Sinnar, Sinnar, Sudan; Station of Medical Essentials (SOME) Institution, Khartoum, Sudan; Faculty of Medicine, Nahda College, Khartoum, Sudan; Station of Medical Essentials (SOME) Institution, Khartoum, Sudan; Faculty of Medicine, Almughtaribeen University, Khartoum, Sudan; Station of Medical Essentials (SOME) Institution, Khartoum, Sudan; Faculty of Medicine, Omdurman Islamic University, Khartoum, Sudan; Department of Oncology, National Cancer Institute, University of Gezira, Wad Madani, Sudan

**Keywords:** breast cancer, diagnosis and treatment interval, early detection, predictors of delay, low- and middle-income countries, Africa

## Abstract

**Background:**

The poor prognosis of breast cancer in Sudan could be due to delayed treatment and diagnosis at an advanced stage. Our study aimed to assess the extent of delays from onset of symptoms to treatment in Sudanese women with breast cancer, as well as identify factors contributing to these delays.

**Materials and Methods:**

We conducted a multi-center cross sectional study between March and April 2023. Data were collected from the medical records and interviews with women with breast cancer in the two main oncology centers in Sudan. Linear regression was used to identify the predictors of delayed presentation.

**Results:**

We interviewed 601 women with breast cancer. The majority of women (50.1%) were diagnosed at locally advanced or metastatic disease. The median interval from the onset of symptoms to receiving oncologic treatment was 221 days (IQR = 92, 496). The longest delay was the presentation delay 61 (31 244) days. The median duration for diagnosis delay and treatment delay was 21 (10.57) days and 27 (10.64) days, respectively. Predictors of early presentation included, being young (β = −5.3; 95% CI = 0.06 to 10), married (β = −264; 95% CI = −427 to −101), divorced (β = −306; 95% CI = −549 to −63), or widowed (β = −320; 95% CI = −-543 to −97), urban residence (β = −107; 95% CI = −213 to −2.3), and seeking traditional healer (β = −204; 95% CI = −383 to −26).

**Conclusion:**

Most Sudanese women with breast cancer experience significant patient delays, often presenting at advanced stages. Factors like being single, older, and living in rural areas contribute to these delays. Increasing breast cancer education, improving healthcare access and addressing sociodemographic barriers can potentially expedite diagnosis and improve outcomes.

## Teaser

The poor prognosis of breast cancer in Sudan could be due to delayed treatment and diagnosis at an advanced stage. This article assesses the extent of delays from onset of symptoms to treatment in Sudanese women with breast cancer and identifies factors contributing to these delays.

**Implications for Practice**

The delay on the patient’s part plays a significant role in the overall delay experienced by Sudanese women with breast cancer. As such, it is vital to establish an early diagnosis strategy that emphasizes raising awareness of the initial signs and symptoms of breast cancer and enhancing access to healthcare services. Furthermore, tackling sociodemographic barriers could potentially facilitate earlier diagnosis and improve outcomes for women with breast cancer.

## Introduction

Breast cancer is the most common cancer globally, with approximately 80% of deaths occurring in low- and middle-income countries (LMICs).^1^ Its incidence rates in Sub-Saharan Africa are on the rise, driven by lifestyle transformations, urbanization, and reduced birth rates.^2,3^ The 5-year survival of breast cancer is more than 90% in high-income countries (HICs); however, it varies in African countries.^4^ In Uganda, it ranges from 35% to 50%, while in Mali it is <20%.^5^ In Sudan, breast cancer is the most common cancer with 5-year survival of 58%.^6^ Low survival rates in sub-Saharan Africa are largely attributable to late-stage at presentation.^3^ Women with breast cancer in Sudan and other African countries are more likely to be diagnosed at advanced stages compared to women in HICs.^7,8^ Up to two-thirds of women with breast cancer in Sudan were diagnosed with stage III or IV^6,9–11^. Early diagnosis and treatment improve breast cancer survival^11^; however, cultural beliefs, cancer stigma and scarcity of screening and diagnostic services contribute to delayed diagnosis in Africa.^2^ There is a lack of data on the extent of delays from onset of symptoms to treatment in Sudanese women with breast cancer, as well as factors contributing to these delays. The Identification these factors would improve early detection and survival rates.

## Materials and methods

### Participants, study design and settings

We conducted a descriptive cross-sectional study including women with breast cancer treated between March and April 2023 at the two main cancer treatment hospitals in Sudan, Khartoum Oncology Hospital and the National Cancer Institute, that offer comprehensive care for cancer patients.

### Data collection tool

A questionnaire was developed based on the study’s objectives and scientific literature. It underwent a review by a panel of experts to ensure its validity and relevance. Suggestions for improvement were implemented. A pilot study involving 31 random patients with breast cancer was conducted to refine the questionnaire based on feedback received. The questionnaire comprises three sections: The sociodemographic information, breast cancer-related traits and the timing of presentation, diagnosis, treatment, and factors that may contribute to delayed intervals. Trained doctors interviewed patients using the Kobo-collect survey tool to ensure quality. Medical records were also reviewed for additional data.

### Patient and system delay’s definitions

Delay occurring in the context of cancer treatment can be categorized into two types: The presentation delay and health system delay. Presentation delay refers to the interval between the onset of symptoms and the initial medical consultation. Health system delay refers to the time between the first medical consultation and the initiation of cancer treatment. Health system delay can be further divided into diagnosis delay, which is the time between initial medical consultation and the histopathologic confirmation, and treatment delay, which is the time between the confirmation of the diagnosis and the initiation of oncologic treatment.^11^

### Statistical analysis

Data were analyzed using the R software version 4.2.2. The Krustal-Wallis test was used to assess the association between delays intervals and the stage and between the diagnosis interval and the type of healthcare personnel visited. A multiple linear regression analysis was used to test the association between presentation delay and different factors. Variables with a *P*-value < .25 in the univariate linear regression were included in the multiple linear regression models. A *P*-value of ≤ .05 was considered statistically significant.

## Results

A total of 601 women with breast cancer gave consent and were interviewed. Table 1 shows characteristics of the study population. Most women had clinical stage III (39.3%), and 55.5 % initially consulted a general surgeon for their breast symptoms (Table 2). The majority (82.9%) stated they did not perform monthly breast self-examination (BSE). Additionally, 82% had limited awareness about the risk factors of breast cancer. However, 61.2% were aware that early treatment improves breast cancer cure rate. Almost all participants (98%) stated they noticed a problem in their breasts themselves (Table 2).

**Table 1.** Sociodemographic characteristics of the study participants.

Variables	*N*	*N* = 601^1^
Name of the cancer hospital	601	
Khartoum Oncology Hospital, Khartoum		312 (51.9%)
National Cancer Institute, Madani		289 (48.1%)
State of origin	601	
Central		340 (56.6%)
Darfur		27 (4.5%)
Eastern		19 (3.2%)
Khartoum		96 (15.9%)
Kordofan		61 (10.1%)
Northern		58 (9.7%)
Age in years	601	49 (40, 56)
Highest level of education	601	
Illiterate		102 (16.9%)
Khalawa^2^		24 (3.9%)
Primary school		188 (31.3%)
Secondary school		176 (29.3%)
University		103 (17.1%)
Above university		8 (1.3%)
Marital status	601	
Single		69 (11.5%)
Married		419 (69.2%)
Divorced		45 (7.5%)
Widowed		68 (11.3%)
Average gross monthly income	601	80 (35, 200)
Health insurance before breast cancer	595	
No		305 (50.7%)
Yes		290 (48.3%)
Missing		6
Sector of residence	601	
Rural		340 (56.6%)
Urban		261 (43.4%)
Age (in years) at first menarche	601	14.00 (13.00, 15.00)
Age (in years) at menopause	389	45.0 (41.0, 49.0)
Unknown		212

^1^ *n* (%); median (IQR).

^2^Khalwa is a form of informal/traditional education where pupils are taught to memorize at least part of Quran as well as the Islamic law and practice.^1^

**Table 2.** Breast cancer-related characteristics of the study participants.

Variables	*N*	*N* = 601^1^
Were you on regular clinical review	601	
No		449 (74.7%)
Yes		152 (25.3%)
Health seeking behavior	601	
I seek immediate medical care		511 (85%)
I seek traditional treatment		59 (9.8%)
I wait to resolve by itself		26 (4.3%)
Others		5 (0.8%)
What was the first treatment you received	601	
Radical mastectomy		268 (44.5%)
Neoadjuvant chemotherapy		310 (51.5%)
Palliative treatment		4 (0.7%)
Hormonal treatment		11 (1.8%)
Others		8 (1.3%)
Self-breast examination monthly	601	
No		498 (82.9%)
Yes		103 (17.1%)
Do you know you are at risk for developing BC	386	
No		318 (82%)
Yes		68 (18%)
Missing		215
Do you know that early seeking of care can cause cancer cure	601	
No		233 (38.8%)
Yes		368 (61.2%)
How did you first find out about this illness	601	
Because of a problem in your breast/ your body that you noticed by yourself		588 (97.8%)
During participation in a screening program		1 (0.2%)
As an incidental finding during a clinical consultation where you had no problem associated with breast		7 (1.2%)
Others		5 (0.8%)
What was the first problem you developed and led to the discovery of this illness	601	
Lump in the breast		434 (72.2%)
Change in the skin over the breast		9 (1.5%)
Change in the nipple of the breast		15 (2.5%)
Discharge from the nipple		26 (4.3%)
An area with harder consistency than usual in the breast		25 (4.2%)
Lumps in the armpit or neck		20 (3.3%)
Pain in the breast or chest or arm		59 (9.8%)
Others		13 (2.2%)
Who was the first healthcare personnel you visited to receive treatment	601	
General practitioner/family doctor		168 (27.9%)
General surgeon		334 (55.5%)
Oncologist/cancer treatment center		58 (9.7%)
Gynecologist		24 (3.9%)
Others		17 (2.8%)
What was her recorded UICC stage of the tumor at the time of diagnosis	601	
Stage 1		22 (3.7%)
Stage 2		192 (31.9%)
Stage 3		236 (39.3%)
Stage 4		67 (11.1%)
Staging is not available		84 (13.9%)
What is her recorded histological type of the tumor	601	
Ductal carcinoma		457 (76.03%)
Lobular carcinoma		61 (10.1%)
Mixed carcinoma (both ductal and lobular)		10 (1.7%)
Medullary carcinoma		16 (2.7%)
Papillary carcinoma		19 (3.2%)
Mucinous (colloid carcinoma)		4 (0.7%)
Tubular carcinoma		2 (0.3%)
Inflammatory carcinoma		9 (1.5%)
Others		23 (3.8%)
Recorded BMI	601	
Underweight		73 (12.1%)
Healthy weight		206 (34.3%)
Overweight		209 (34.7%)
Obese		113 (18.8%)

^1^ *n* (%).

**Figure 1.**
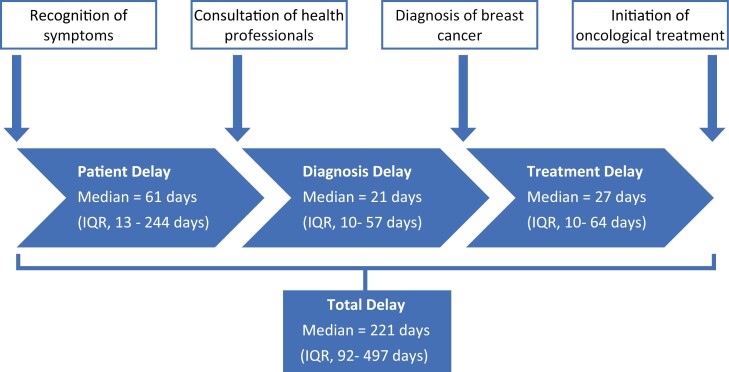
Average intervals of presentation, diagnosis, and treatment.

The median duration of the overall delay was 221 days (IQR, 92-496). The longest delay was the presentation delay 61 (IQR, 31-244) days (Figure 1). The independent predictors of early presentation were being young, married, divorced, or widowed, urban residence, seeking traditional healer (Table 3).

**Table 3.** Linear regression of socio-demographic data and breast cancer presentation interval.

	Univariate			Multivariate		
Characteristic	Beta	95% CI	*P*-value	Beta	95% CI	*P*-value
Age (years)	4.5	−0.11, 9.2	.056	5.3	0.06, 10	.047
Highest level of education						
Illiterate	—	—				
Khalawa	−125	−409, 160	.4	—	—	—
Secondary school	−32	−186, 123	.7	—	—	—
Primary school	−86	−242, 70	.3	—	—	—
University	−197	−372, −22	**.028**	—	—	—
Above university	−138	−598, 322	.6	—	—	—
Marital status						
Single	—	—		—	—	
Married	−259	−421, −97	**.002**	−264	−427, −101	**.002**
Divorced	−264	−503, −26	**.030**	−306	−549, −63	**.014**
Widowed	−240	−453, −27	**.027**	−320	−543, −97	**.005**
Average gross monthly income	0.00	−0.01, 0.01	>.9	—	—	—
Health insurance before this illness						
No	—	—				
Yes	−35	−138, 68	.5	—	—	—
Sector of residence						
Rural	—	—		—	—	
Urban	−127	−230, −25	**.015**	−107	−213, −2.3	**.045**
Were you on regular clinical review						
No	—	—		—	—	
Yes	92	−25, 210	.12	54	−70, 179	.4
Health seeking behavior						
I seek immediate medical care	—	—		—	—	
I seek traditional treatment	−173	−345, −0.56	**.049**	−204	−383, −26	**.025**
I wait to resolve by itself	43	−209, 295	.7	17	−233, 266	.9
Others	−231	−794, 332	.4	−204	−764, 356	.5
Self-breast examination monthly						
No	—	—		—	—	
Yes	−80	−216, 55	.2	−23	−168, 122	.8
Do you know you are at risk for developing BC						
No	—	—				
Yes	−101	−282, 79	.3	—	—	—
Do you know that early seeking of care can cause cancer cure						
No	—	—		—	—	
Yes	−107	−212, −2.2	**.045**	−88	−203, 28	.14
How did you first find out about this illness						
Because of a problem in your breast/ your body that you noticed by yourself	—	—				
During participation in a screening program	−277	−1,533, 980	.7	—	—	—
As an incidental finding during a clinical consultation where you had no problem associated with breast	−161	−639, 316	.5	—	—	—
Others	−252	−816, 312	.4	—	—	—
What was the first problem you developed and led to the discovery of this illness						
Lump in the breast	—	—				
Change in the skin over the breast	−67	−490, 355	.8	—	—	—
Change in the nipple of the breast	−169	−499, 160	.3	—	—	—
Discharge from the nipple	−47	−300, 206	.7	—	—	—
An area with harder consistency than usual in the breast	−69	−327, 190	.6	—	—	—
Lumps in the armpit or neck	−81	−368, 206	.6	—	—	—
Pain in the breast or chest or arm	−176	−350, −1.6	**.048**	—	—	—
Others	−229	−582, 124	.2	—	—	—
Who was the first healthcare personnel you visited to receive treatment						
General practitioner/family doctor	—	—		—	—	
General Surgeon	92	−22, 215	.13	51	−69, 170	.4
Oncologist/cancer treatment center	−81	−272, 109	.4	−44	−237, 148	.7
Gynecologist	−101	−374, 172	.5	−77	−349, 195	.6
Others	168	−150, 487	.3	193	−123, 509	.2

Bold values denote statistical significance at the P<0.05 level.

The association between clinical stages and categories of delays is shown in Table 4. The only statistically significant difference was observed between the median the stages (*P* < .001), with stage 1 having the longest diagnosis interval (49 days), followed by stage 4 (34 days), then stage 2 (19 days) and stage 3 (19 days).

**Table 4.** Median presentation, diagnosis, treatment, and overall intervals by UICC stage at time of diagnosis (*N* = 601).

Characteristic	Overall, Md (IQR)	Stage 1, *N* = Md (IQR)	Stage 2 Md (IQR)	Stage 3 Md (IQR)	Stage 4, Md (IQR)	Staging is not available Md (IQR)	*P*-value^1^
Presentation interval	61 (13, 244)	31 (14, 86)	65 (11, 365)	77 (15, 243)	31 (14, 198)	38 (10, 157)	.11
Diagnosis interval	21 (10, 57)	49 (20, 163)	19 (8, 39)	19 (8, 48)	34 (17, 93)	33 (12, 60)	**<.001**
Treatment interval	27 (10, 64)	25 (8, 94)	24 (9, 54)	26 (9, 70)	31 (11, 59)	35 (17, 70)	.2
Total interval	221 (92, 496)	289 (160, 373)	218 (98, 626)	230 (96, 444)	220 (96, 623)	183 (80, 356)	.4

^1^Kruskal-Wallis rank sum test.

Abbreviations: Md, median; IQR, interquartile range. Bold values denote statistical significance at the P < 0.05 level

**Figure 2.**
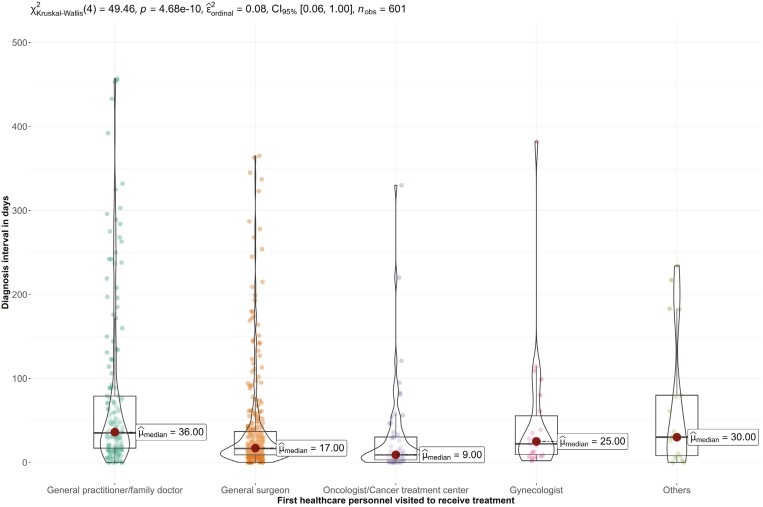
Diagnosis interval in relation to the first healthcare personnel visited by the participants.

The healthcare personnel initially visited by the participants significantly affected the median diagnosis interval (*P* < .001). The longest median interval was 36 days for general practitioner/family doctor, while the shortest was 9 days for oncologists (Figure 2).

The frequently reported reasons for patients delay included underestimating breast symptoms and not considering the possibility of having breast cancer (31.6%), not being bothered by breast lesion (27.3%), lack of awareness about the availability of curative treatment for breast cancer (7%), lack of knowledge about cancer (7%), and misinterpreting symptoms due to previous history of benign breast condition (3%) (Supplementary Table S1).

The leading cause of delayed diagnosis was the failure of doctors to suspect cancer at initial consultation (7.5%). This was followed by delays in receiving biopsy results (4.7%), financial challenges (4.7%), and fear related to the receiving of a cancer diagnosis (4.7%) (Supplementary Table S2). Fear of undergoing cancer treatment was the main cause (14%) of delayed treatment, followed by financial challenges (5.2%) (Supplementary Table S3).

## Discussion

Our findings show that the median interval between first discovery of breast symptoms and treatment initiation exceeded 18 months, which was long enough to lower the survival. The greatest delay occurred between symptom discovery and first presentation, with a median of 61 days. This is longer than the 30 days reported in other LMICs,^12^ Mexico (10 days), South Africa (23 days), and Iran (30 days),^13,14^ but shorter than Pakistan (255 days), Brazil (127 days), and Malaysia (90 days).^15-17^ While cancer control programs typically aim for a patient delay of 30 or 14 days,^13,18^ the absence of a national breast cancer screening program in Sudan makes achieving this goal challenging.^19^ The frequently reported reasons for patients delay in this study were underestimating breast symptoms, lacking awareness about breast cancer and misinterpreting symptoms. Similar reasons were reported in LMICs.^12^ This highlights the needs for early diagnosis approach focusing on the promotion of the awareness of early signs and symptoms among the public.

We found that married women were more likely to seek medical care earlier than unmarried women, a finding that is in lines with a previous meta-analysis.^20^ We also observed that older women and those residing rural areas experienced significant longer delay in seeking medical care for breast symptoms, a trend that aligns with a study from Ethiopia.^21^ This delay be attributed to older women perceive breast changes as a normal postmenopausal phenomenon. These finding underscore the importance of breast cancer awareness campaigns, particularly for those at higher risk. The use of trained volunteers to conduct clinical breast examinations in rural area of Sudan has been proven to be a simple yet effective strategy for early symptoms detection.^22^

In our study, 98% of breast cancer cases were self-detected by the women, a figure slightly higher than the 90% reported in Ethiopia.^23^ This underscores the importance of BSE as a primary mechanism for breast cancer detection. However, only 17% of our study participants perform BSE monthly, a rate comparable to that reported across Africa.^24^ Therefore, it is vital to enhance women’s practice of BSE by promoting early detection programs that focus on improving the technique and frequency of BSE.

In this study, women with breast abnormalities tend to seek medical help when they experience pain, similar to previous studies.^25,26^ It is important to note that pain may not be the only present symptom for breast cancer, rather the symptom drives patients to seek medical care.

The median duration for both diagnosis and treatment delays in this study was deemed satisfactory, falling within the recommended 1-month time frame.^18^ several factors contributed to these delays, including fear of cancer treatment, financial challenges, delays in receiving biopsy results and the social stigma associated with cancer diagnosis. Furthermore, limited access to mammography and image-guided biopsy is pose a barrier to early diagnosis in Sudan^19^ . Misinterpretation of symptoms and misdiagnosis by doctors were also significant factors leading to prolonged intervals in Africa.^12^

We observed an association between health system delay and the medical specialty of the provider that was consulted, with the longest interval being for general practitioners. This is particularly relevant in resource-limited setting where the majority of patients first contact a general practitioner, who may not be familiar with breast cancer screening and diagnostic guidelines. Therefore, educating general practitioners and improving referral procedures are crucial to facilitate the early diagnosis and treatment of breast cancer.

Our study reveals an association between cancer stage and diagnosis interval, with the longest diagnostic delay observed in stage 1 compared to other stages. This delay in diagnosing small tumors could be attributed to the limited access to diagnostic services and specialized physicians.^19^ However, previous studies examining the associations between delay and tumor stage have yielded inconsistent results.^11,27^

This study focuses on the delay in the context of breast cancer management, a topic that has not been widely studied in LMICs. It took place in Sudan’s two main cancer hospitals, making it a representative sample of Sudanese women with breast cancer. However, it is important to acknowledge the limitations of this study, including the potential for recall bias.

## Conclusion

The majority of Sudanese women with breast cancer are receiving treatment at advanced stages after significant delays. The patient’s delay plays a crucial role in this overall delay. Key predictors of this delay include being single, older, and residing in a rural area. There is a pressing need for more education on breast cancer, particularly for those at higher risk. Furthermore, enhancing access to healthcare services, especially in non-urban areas, and addressing sociodemographic barriers could potentially facilitate earlier diagnosis and improve outcomes.

## Supplementary Material

oyae066_suppl_Supplementary_Table_1

oyae066_suppl_Supplementary_Table_2

oyae066_suppl_Supplementary_Table_3

## Data Availability

The data underlying this article will be shared on reasonable request to the corresponding author.

## References

[1] WHO launches new roadmap on breast cancer. https://www.who.int/news/item/03-02-2023-who-launches-new-roadmap-on-breast-cancer

[2] Anyigba CA , Awandare GA, Paemka L. Breast cancer in sub-Saharan Africa: the current state and uncertain future. Exp Biol Med (Maywood). 2021;246(12):1377-1387. 10.1177/15353702211006047 33926257 PMC8243219

[3] Sung H , Ferlay J, Siegel RL, et al. Global Cancer Statistics 2020: GLOBOCAN Estimates of Incidence and Mortality Worldwide for 36 Cancers in 185 Countries. CA Cancer J Clin.s. 2021 71(3):209-249. 10.3322/caac.21660 33538338

[4] Allemani C , Matsuda T, Di Carlo V, et al; CONCORD Working Group. Global surveillance of trends in cancer survival 2000-14 (CONCORD-3): analysis of individual records for 37 513 025 patients diagnosed with one of 18 cancers from 322 population-based registries in 71 countries. Lancet. 2018;391(10125):1023-1075. 10.1016/S0140-6736(17)33326-3 29395269 PMC5879496

[5] McCormack V , McKenzie F, Foerster M, et al. Breast cancer survival and survival gap apportionment in sub-Saharan Africa (ABC-DO): a prospective cohort study. The Lancet Global Health. 2020;8(9):e1203-e1212. 10.1016/S2214-109X(20)30261-8 32827482 PMC7450275

[6] Muddather HF , Elhassan MMA, Faggad A. Survival outcomes of breast cancer in Sudanese women: a hospital-based study. JCO Glob Oncol. 2021;7(7):324-332. 10.1200/GO.20.00538 33617296 PMC8081542

[7] Olayide A , Isiaka A, Ganiyu R, et al. Demographic pattern, tumor size and stage of breast cancer in Africa: a meta-analysis. Asian Pacific J Cancer Care. 2021;6(4):477-492. 10.31557/apjcc.2021.6.4.477-492

[8] Elgaili EM , Abuidris DO, Rahman M, Michalek AM, Mohammed SI. Breast cancer burden in central Sudan. Int J Womens Health. 2010;2:77-82. 10.2147/ijwh.s8447 21072300 PMC2971742

[9] Saad EA , Khair R. Clinico-pathological features of breast cancer in patients below 50 years of age inKhartoum. Khartoum Medical Journal. 2016; 8(3):1158-1163.

[10] Mariani-Costantini, R., Elhassan, M. M. A., Aceto, G. M., Mohamedani, A. A., & Awadelkarim, K. D. (2017). Epidemiology, Pathology, Management and Open Challenges of Breast Cancer in Central Sudan: A Prototypical Limited Resource African Setting. In P. V. Pham (Ed.), Breast Cancer (Chapter 1). IntechOpen. 10.5772/67175

[11] Caplan L. Delay in breast cancer: implications for stage at diagnosis and survival. Front Public Health. 2014;2:87. 10.3389/fpubh.2014.00087 25121080 PMC4114209

[12] Brand NR , Qu LG, Chao A, Ilbawi AM. Delays and barriers to cancer care in low- and middle-income countries: a systematic review. Oncologist. 2019;24(12):e1371-e1380. 10.1634/theoncologist.2019-0057 31387949 PMC6975966

[13] Moodley J , Cairncross L, Naiker T, Constant D. From symptom discovery to treatment - women’s pathways to breast cancer care: a cross-sectional study. BMC Cancer. 2018;18(1):312. 10.1186/s12885-018-4219-7 29562894 PMC5863383

[14] Bright K , Barghash M, Donach M, et al. The role of health system factors in delaying final diagnosis and treatment of breast cancer in Mexico City, Mexico. The Breast. 2011;20(Suppl. 1):S54-S59. https://doi.org/10.1016/j.breast.2011.02.012 21371885 10.1016/j.breast.2011.02.012

[15] Soh JY , Yahya MM, Bachok N, et al. Factors associated with delay in seeking care for breast symptoms. BMC Womens Health. 2022;22(1):316. 10.1186/s12905-022-01898-5 35897099 PMC9331147

[16] Medeiros GC , Thuler LCS, Bergmann A. Determinants of delay from cancer diagnosis to treatment initiation in a cohort of Brazilian women with breast cancer. Health Soc Care Commun. 2021;29(6):1769-1778. https://onlinelibrary.wiley.com/doi/abs/10.1111/hsc.13284 10.1111/hsc.13284 33438787

[17] Majeed I , Ammanuallah R, Anwa AW, Rafique HM, Imran F. Diagnostic and treatment delays in breast cancer in association with multiple factors in Pakistan. East Mediterr Health J. 2021;27(1):23-32. 10.26719/emhj.20.051 33538316

[18] Cancer Council Victoria and Department of Health Victoria. Optimal care pathway for people with breast cancer, 2nd edn 2021. Cancer Council Victoria, Melbourne. https://www.cancer.org.au/assets/pdf/breast-cancer-2nd-edition

[19] Husain NE , Burhan A, Ahmed IAI, Mohammed SI, Hammad N. Women’s cancers in Sudan with a focus on cervical cancer: turmoil, geopolitics and opportunities. 2022. http://ecancer.org/en/journal/article/1433-womens-cancers-in-sudan-with-a-focus-on-cervical-cancer-turmoil-geopolitics-and-opportunities 10.3332/ecancer.2022.1433 PMC9470168 36200016

[20] Yuan R , Zhang C, Li Q, Ji M, He N. The impact of marital status on stage at diagnosis and survival of female patients with breast and gynecologic cancers: a meta-analysis. Gynecol Oncol. 2021;162(3):778-787. 10.1016/j.ygyno.2021.06.008 34140180

[21] Hassen AM , Hussien FM, Asfaw ZA, Assen HE. Factors Associated with Delay in Breast Cancer Presentation at the Only Oncology Center in North East Ethiopia: A Cross-Sectional Study. J Multidiscip Healthc. 2021;14:681-694. 10.2147/JMDH.S301337. 33776446 PMC7989045

[22] Abuidris DO , Elsheikh A, Ali M, et al. Breast-cancer screening with trained volunteers in a rural area of Sudan: a pilot study. Lancet Oncol. 2013;14(4):363-370. 10.1016/S1470-2045(12)70583-1 23375833

[23] Kebede L , Abdo M, Megerso A. Breast self-examination practice and associated factors among women of reproductive age in Adama Town, Oromia Regional State, Ethiopia. Central African J Public Health. 2021;7(4):227-235. 10.11648/j.cajph.20210704.22

[24] Seifu W , Mekonen L. Breast self-examination practice among women in Africa: a systematic review and Meta-analysis. Arch Public Health. 2021;79(1):149. 10.1186/s13690-021-00671-8 34419150 PMC8379892

[25] Agbeko AE , Arthur J, Bayuo J, Kaburi BB, Kyei I. Seeking healthcare at their “right” time; the iterative decision process for women with breast cancer. BMC Cancer. 2020;20(1):1011. 10.1186/s12885-020-07520-x 33076850 PMC7574193

[26] Ng DY , Car LT, Ng MJM, et al. Identifying barriers to early presentation in patients with locally advanced breast cancer (LABC) in Northern Singapore: Qualitative study. PLoS One. 2021;16(5):e0252008. https://journals.plos.org/plosone/article?id=10.1371/journal.pone.0252008 34032802 10.1371/journal.pone.0252008 PMC8148318

[27] Eltayeb MA , Faggad A, Abbadi OS, Elhassan MMA. Characteristics of breast cancer at first presentation in sudanese patients attending the National Cancer Institute–University of Gezira (NCI–UG). *Arch Breast Cancer*. 2020;7(3):104-110. 10.32768/abc.202073104-110

