# Supplementary material for: Delays in presentation, diagnosis, and treatment in Sudanese women with breast cancer: a cross-sectional study
Source: Oncologist. 2024 Apr 20;29(6):e771–8. doi: 10.1093/oncolo/oyae066 (PMC11144982; doi:10.1093/oncolo/oyae066)
Supplement: oyae066_suppl_Supplementary_Table_1 [file oyae066_suppl_supplementary_table_1.docx]

| **Supplementary Table 1: Reasons provided by breast cancer patients for presentation delay** |  |
| --- | --- |
| **Variables*** | **N = 601**^1^ |
| 1. The symptoms didn't bother me | 164 (27.3%) |
| 2. I don't know what cancer was | 35 (5.8%) |
| 3. I don't think it might be cancer | 190 (31.6%) |
| 4. The presence of pain reassured me | 14 (2.3%) |
| 5. I was worried it might be contagious | 19 (3.2%) |
| 6. Because I had a previous diagnosis of a benign breast condition, so I thought it could be the same | 18 (3%) |
| 7. I didn't know where an appropriate medical facility was | 1 (0.2%) |
| 8. I know someone with a similar symptoms who had a bad experience at the hospital before | 7 (1.2%) |
| 9. I tried a traditional healer or prayer first | 15 (2.5%) |
| 10. I can't afford the financial cost of the medical care | 22 (3.%) |
|  |  |
| 11. I was afraid of the treatment including surgery or chemotherapy | 24 (4%) |
| 12. I didn't know that there is an effective treatment | 4 (7%) |
| 13. I didn't want anyone to know that I had a breast problem (stigma) | 10 (1.7%) |
| 14. My husband or my family didn't give me permission to go to the hospital | 3 (0.5%) |
| 16. I was too busy at my home or my job to go to the doctor | 24 (4%) |
| 17. nothing applies to me | 273 (45.5%) |
| ^1^n (%)  *multiple answer question | |
