# Supplementary material for: Delays in presentation, diagnosis, and treatment in Sudanese women with breast cancer: a cross-sectional study
Source: Oncologist. 2024 Apr 20;29(6):e771–8. doi: 10.1093/oncolo/oyae066 (PMC11144982; doi:10.1093/oncolo/oyae066)
Supplement: oyae066_suppl_Supplementary_Table_2 [file oyae066_suppl_supplementary_table_2.docx]

| **Supplementary table 2. Reasons provided by breast cancer patients for diagnosis delay** |  |
| --- | --- |
| **Variables** | **N = 601**^1^ |
| 1. The doctor was not concerned and did not refer me to further evaluation | 45 (7.5%) |
| 2. My history of previous benign breast condition caused the doctor to reassure me | 15 (2.5%) |
| 3. I was referred, but I was afraid of further medical evaluation | 17 (2.8%) |
| 4. I was referred, but there was a long waiting time because of the capacity challenge | 10 (1.7%) |
| 5. I was afraid if I had a biopsy, it could cause the cancer to spread | 1 (0.2%) |
| 6. I did a biopsy, but the results took a longer time | 28 (4.7%) |
| 7. I underwent breast-specific investigations that were 0t interpreted accurately, so I was inappropriately reassured | 4 (0.7%) |
| 8. I saw more than one healthcare provider who treated me for an infection or problem other than cancer first | 7 (1.2%) |
| 9. 0n lump symptoms at presentation | 8 (1.3%) |
| 10. nothing applies to me | 471 (78%) |
| 11. Financial issues | 28 (4.7%) |
| ^1^n (%) | |
