# Supplementary material for: Delays in presentation, diagnosis, and treatment in Sudanese women with breast cancer: a cross-sectional study
Source: Oncologist. 2024 Apr 20;29(6):e771–8. doi: 10.1093/oncolo/oyae066 (PMC11144982; doi:10.1093/oncolo/oyae066)
Supplement: oyae066_suppl_Supplementary_Table_3 [file oyae066_suppl_supplementary_table_3.docx]

| **Supplementary table 3. Reasons provided by breast cancer patients for treatment delay** |  |
| --- | --- |
| **Variables** | **N = 601**^1^ |
| 1.fear of cancer treatment | 84 (14%) |
| 2. Fear of partner abandonment regarding the cosmetic effects of treatment | 7 (1.2%) |
| 3. Self-fear of the cosmetic effects | 15 (2.5%) |
| 4. I can't afford the financial cost of the medical treatment | 31 (5.2%) |
| 5.Fears of social embarrassment regarding the treatment of disease | 20 (3.3%) |
| 6. inaccessibility to healthcare | 18 (3.0%) |
| 7. others | 33 (5.5%) |
| 8. 0thing applies to me | 439 (73%) |
| 9. other medical conditions that cause a delay in the provision of definitive treatment | 11 (1.8%) |
| ^1^n (%) | |
